# Dataset of Harvard Step Test Results for Assessing Cardiovascular Fitness in Female Cricketers in Bangladesh

**DOI:** 10.1016/j.dib.2025.111449

**Published:** 2025-03-06

**Authors:** Farjana Akter Boby, Marzia Ahmed, Imran Mahmud

**Affiliations:** 1 Dept. of Physical Education & Sports Science, Faculty of Health and Life Science, Daffodil International University, Daffodil Smart City, Ashulia, 1341, Dhaka, Bangladesh; 2 Department of Software Engineering, Daffodil International University, Daffodil Smart City, Ashulia, 1341, Dhaka, Bangladesh

**Keywords:** Physical endurance metrics, Pulse Recovery Rates, Female Athletes Perfor5mance, Sports Physiology, Athlete health assessment

## Abstract

The dataset consists of results of the Harvard Step Test conducted on female cricketers participating in the Bangladesh National Women's Cricket League 2021–22, which is the major competition level for women cricketers in Bangladesh. The data was collected by eight coaches representing the eight divisions of Bangladesh in the year 2022. The data set contains demographic information: age, height, weight, BMI; years of playing experience; resting heart rates; pulse recovery rates at specified intervals after the test (1.5 minutes, 2.5 minutes, 3.5 minutes); and fitness scores computed using a standard formula. Data was collected through a controlled experimental design to ensure uniformity in administering the tests. Participants went through the standard Harvard Step Test under supervised conditions in order to determine cardiovascular endurance. The dataset is large, robust, and organized to be useful for analyses and studies in the area of cardiovascular fitness, athletic performance, or long-term athlete health. It can also be used in designing training programs, understanding recovery profiles, and setting fitness standards for female athletes. It would be of importance to scholars in the sports sciences, public health, and exercise physiology because it fills some of the gaps in available data on female athletes from South Asia.

Specifications Table

**Table:** 

Subject	Health and Medical sciences (Sports Sciences)
Specific subject area	Cardiovascular fitness assessment of female cricketers using the Harvard Step Test
Type of data	Table, Chart, Graph Raw, Analysed.
Data collection	Data were collected using the Harvard Step Test during the 2021–22 Bangladesh National Women's Cricket League. Standardized testing procedures were followed for all measurements of physical characteristics (age, height, weight) and physiological responses (resting heart rate, pulse recovery rates). Testing was conducted by eight coaches from across Bangladesh. Fitness scores were calculated using the standardized formula of the Harvard Step Test. The inclusion criteria were all female cricketers 16 years of age or older. Alternatively, exclusion criteria included the presence of incomplete tests, or illness/disease compromising participation.
Data source location	Bangladesh National Women's Cricket League 2021–22 (8 divisions across Bangladesh). Data stored at Mendeley Repository.
Data accessibility	Repository name: Mendeley Data Data identification number: 10.17632/hnv22pyt4f.3 Direct URL to data: https://data.mendeley.com/datasets/hnv22pyt4f/3 Instructions for accessing these data: Publicly available under a CC-BY license.
Related research article	None.

## 1 Value of the Data

• Unique Dataset for Female Athlete Fitness in South Asia: This dataset is unique as only a few focus on female cricketers in Bangladesh. It will present very rare insights into the heart fitness of top athletes within a developing country's sports setting. It will be useful for setting fitness standards for women in cricket and other sports in similar areas.
• Support for Cross-disciplinary Research: This dataset can be used by researchers in sports science, public health, and sports psychology to examine in more detail the patterns of heart health, recovery times, and their links with athletic performance measures in this varied group of female athletes.
• Foundation for Training and Rehabilitation Programs: The information can help create training plans and recovery methods based on the specific physical needs of female cricketers.
• Contribution to Longitudinal Studies: This data set can be merged with future data sets to examine trends in fitness levels over time, especially as training methods and competition intensity change.
• Reusability in Machine Learning and Predictive Modeling: The dataset is organized, and the variables are clear, making it good for predictive analysis, like predicting fitness scores or recovery times based on player profiles.
• Generalizability to Similar Populations: Although this data is specific to Bangladesh, it can be used to find similarities with other underrepresented groups in global sports science. This helps promote inclusive research.

## 2 Background

The data [1] were collected for cardiovascular fitness among female cricketers participating in the Bangladesh National Women's Cricket League 2021–22. This is the premier competition for women cricketers in the country. There was a motivation due to the general unavailability of public fitness data regarding female athletes from South Asia, and particularly cricket, which is one of the fastest-growing female sports in Bangladesh.

Lucien Brouha [2] introduced the Harvard Step Test during World War II as a simple and effective method to evaluate cardiovascular fitness and recovery rates. This method has been widely validated and adapted in various research settings. Walid Soliman Ismail Mahmoud Elsaidy et al. [3] analyzed the validity and reliability of the Harvard Step Test and its variants (like KAJHST) for estimating VO2 max compared to direct laboratory measurements. The study highlights the test's applicability in predicting fitness levels with strong correlation coefficients. (Journal of Applied Sports Science, 2021).

Sandstedt et al. [4] investigated the use of modified versions of the Harvard Step Test in populations with specific health conditions, demonstrating its flexibility and relevance across various demographics and fitness levels. (Pediatric Rheumatology, 2013). Toumpakari et al. applied a modified Harvard Step Test to analyze [5] cardiometabolic risk factors in adolescents. This study showcases how step tests can be adapted for diverse populations while maintaining their efficacy in fitness evaluation. In the context of evaluating dynamic stability and muscular responses during physical activities, prior studies, such as one investigating suspended lunges under varying stability conditions, provide valuable insights. This research [6] demonstrated high reliability (ICC: 0.821–0.970) in biomechanical measurements, with significant positive correlations (r = 0.393–0.826) between muscle activity and body center of mass acceleration across exercise variations. These findings underscore the role of stability and muscle engagement in advanced athletic performance.

The Harvard Step Test was selected for the assessment of cardiovascular endurance and recovery because it is one of the most widely used, reliable, and inexpensive tests. A total of eight regional coaches collected the data to make the representation complete in all divisions of Bangladesh. The parameters recorded included height, weight, BMI, resting heart rate, pulse recovery rates, and calculated fitness scores.

A thorough search on Google Scholar and other sources did not reveal any publicly available datasets on cardiovascular fitness specifically for female cricketers. Most existing studies focus on general athlete fitness or mixed-gender samples. This dataset is unique in assessing cardiovascular endurance among female cricketers using the Harvard Step Test, providing detailed pulse recovery data and fitness scores from national-level athletes in Bangladesh. It offers a valuable resource for benchmarking and comparative analysis, filling a gap in cricket-specific fitness research.

This dataset provides a foundation for researchers in sports science, coaches, and policymakers to understand the fitness levels of elite female cricketers. It can also help inform training, performance optimization, and health monitoring programs for athletes. The dataset is independent of any specific research publication and is intended as a standalone resource for reuse in scientific and practical applications.

## 3 Data Description

The dataset is available in a Microsoft Excel file titled “Data set of Harvard Step Test.xlsx” and contains one main sheet. We have collected data from 103 female cricketers from Bangladesh National Women's Cricket League. Following is the structure of the dataset, which explains the columns and their significance (Table 1).

**Table 1:** Overview of Columns in the Dataset.

Column Name	Description	Data Type	Example Values
SL	Serial number of participants	Integer	1, 2, 3
Name	Name of the cricketer	Text	Marzia Ahmed
Age	Age of the participant in years	Integer	16, 17
Height (Meter)	Height of the participant in meters	Float	1.52, 1.58
Weight (KG)	Weight of the participant in kilograms	Integer	46, 52
BMI	Body Mass Index, calculated using the formula: Weight (kg) / [Height (m)]²	Float	19.91, 20.83
Playing Age	Number of years the participant has been actively playing cricket	Integer	3, 4, 7
Resting Heart Rate	Participant's heart rate (beats per minute) measured before the test	Integer	70, 72
Duration (minutes)	Duration of the Harvard Step Test (usually 5 minutes)	Text/Integer	5 min
PR1 (1–1.5min)	Pulse rate (beats per minute) recorded 1–1.5 minutes after completing the Harvard Step Test	Integer	85, 90
PR2 (2–2.5min)	Pulse rate (beats per minute) recorded 2–2.5 minutes after completing the Harvard Step Test	Integer	78, 82
PR3 (3–3.5min)	Pulse rate (beats per minute) recorded 3–3.5 minutes after completing the Harvard Step Test	Integer	70, 74
Fitness Score	Calculated based on the standard Harvard Step Test formula	Float	77.32, 96.77

**Structure of the File:** filename= Data set of Harvard Step Test.xlsx

Sheet Name: Test Score

Data Type: Tabular data containing raw and calculated metrics.

**Folder/Subfolder Structure for Dataset Repository**

**1. Root Folder**

• README.txt: A detailed description of the dataset, including purpose, methods, and data format.
• Data set of Harvard Step Test.xlsx: The main dataset containing all variables.
• Python Code: The data visualization codes has been includes also.

**2. Subfolder: Figures**

• Includes illustrative figures of data distribution or trends (such as histograms or scatterplots, pair plot, box plot or correlation).

### 3.1 Visualization

**Fig. 1:**
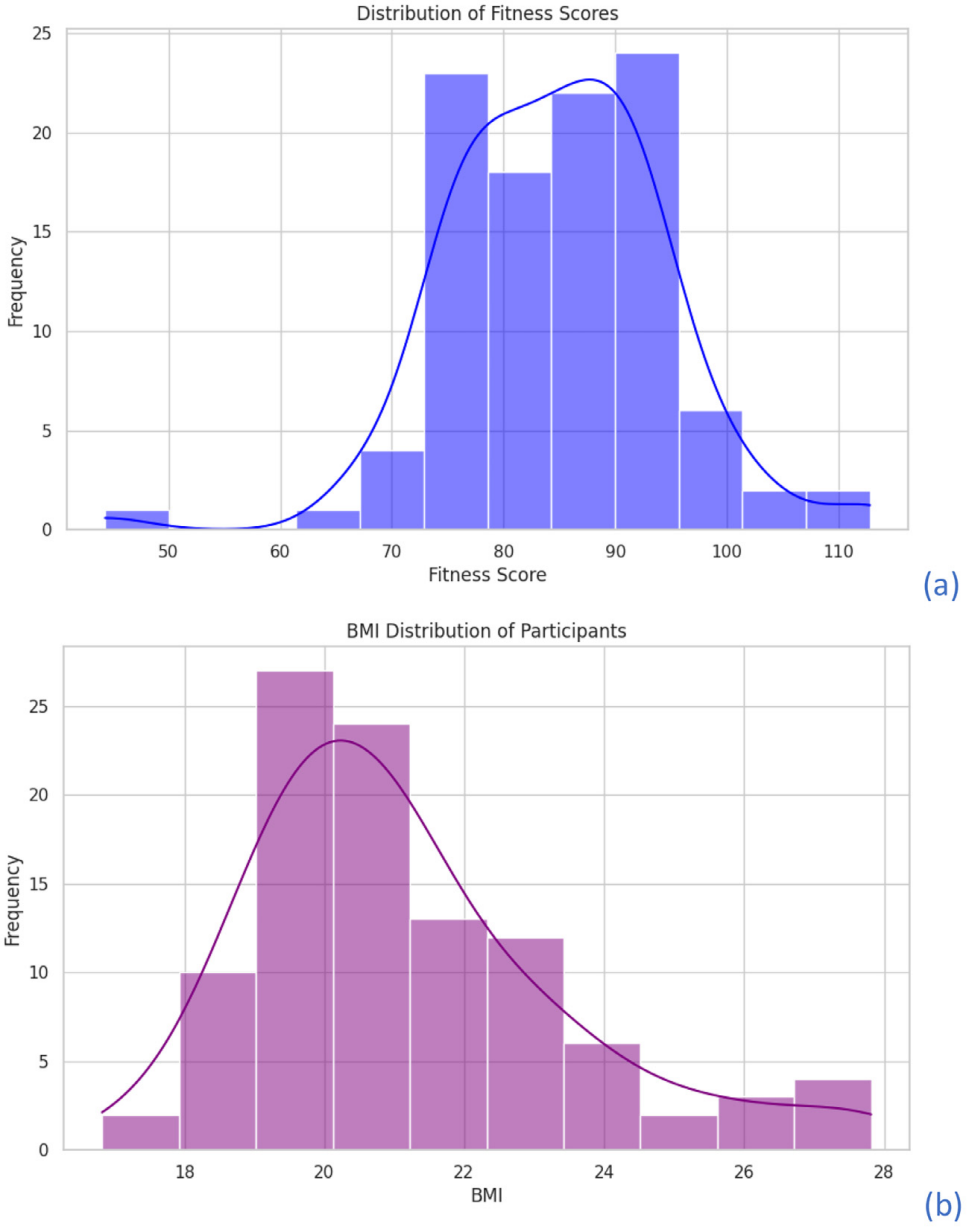
(a) Histogram of Fitness Scores (b) Histogram of BMI Distribution.
• This scatterplot Fig. 2, visualizes the relationship between fitness score and BMI, illustrating potential trends between these two variables. It helps to identify any patterns or correlations, showing how BMI may impact the fitness score.

• A histogram Fig. 1(a) showing the distribution of fitness scores across all participants. This visualization highlights the variations in cardiovascular fitness levels and gives insight into the general fitness trends within the dataset. In Fig. 1, the Y-axis represents the count of participants within each fitness category based on their Harvard Step Test scores. The distribution illustrates the number of female cricketers falling into different fitness levels rather than a percentage-based representation. This helps in understanding the absolute frequency of fitness scores among the sampled athletes. Fig.: 1 (b) This plot shows the overall range of BMI values, which can be used to assess the general body composition and categorize participants based on their BMI levels.

**Fig. 2:**
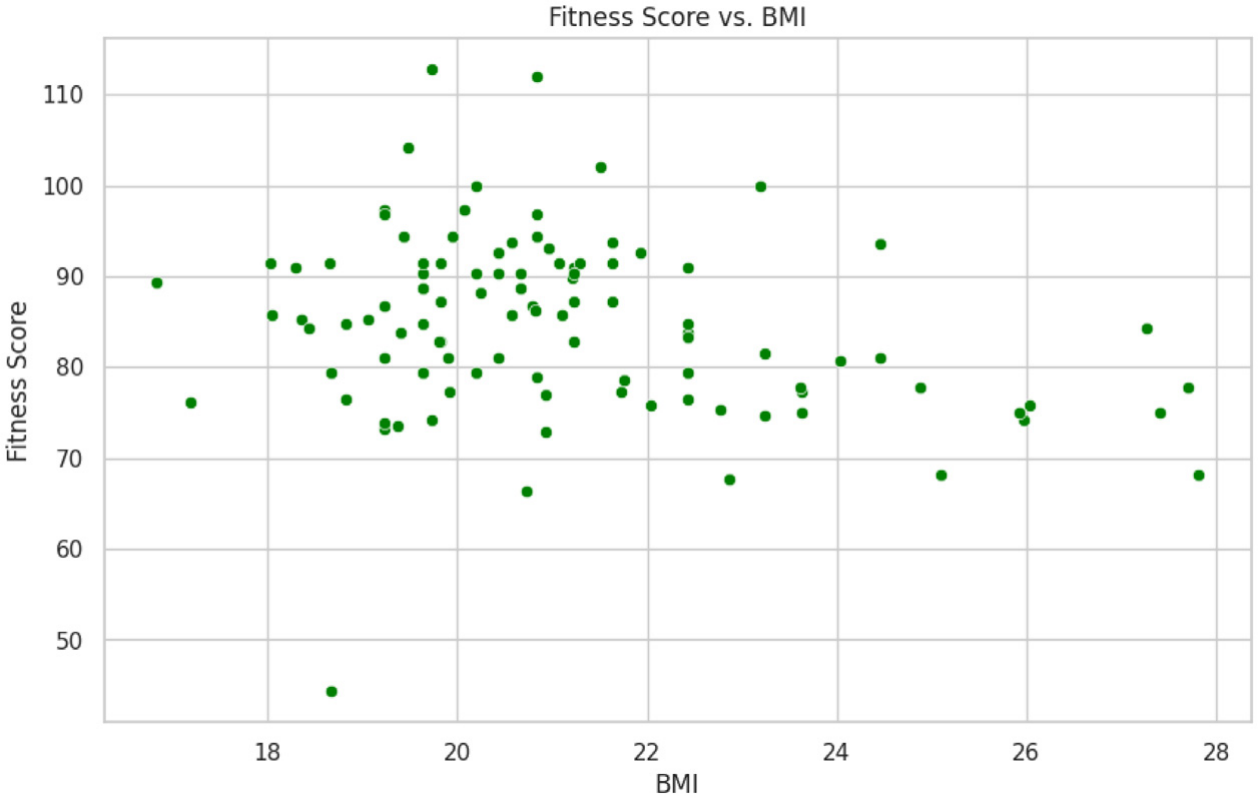
Scatterplot of Fitness Score vs. BMI.
• A box plot Fig. 3 displaying the variation in fitness scores across different age groups. The plot provides insight into the distribution and outliers of fitness scores by age, helping to understand how age might influence physical fitness levels.

**Fig. 3:**
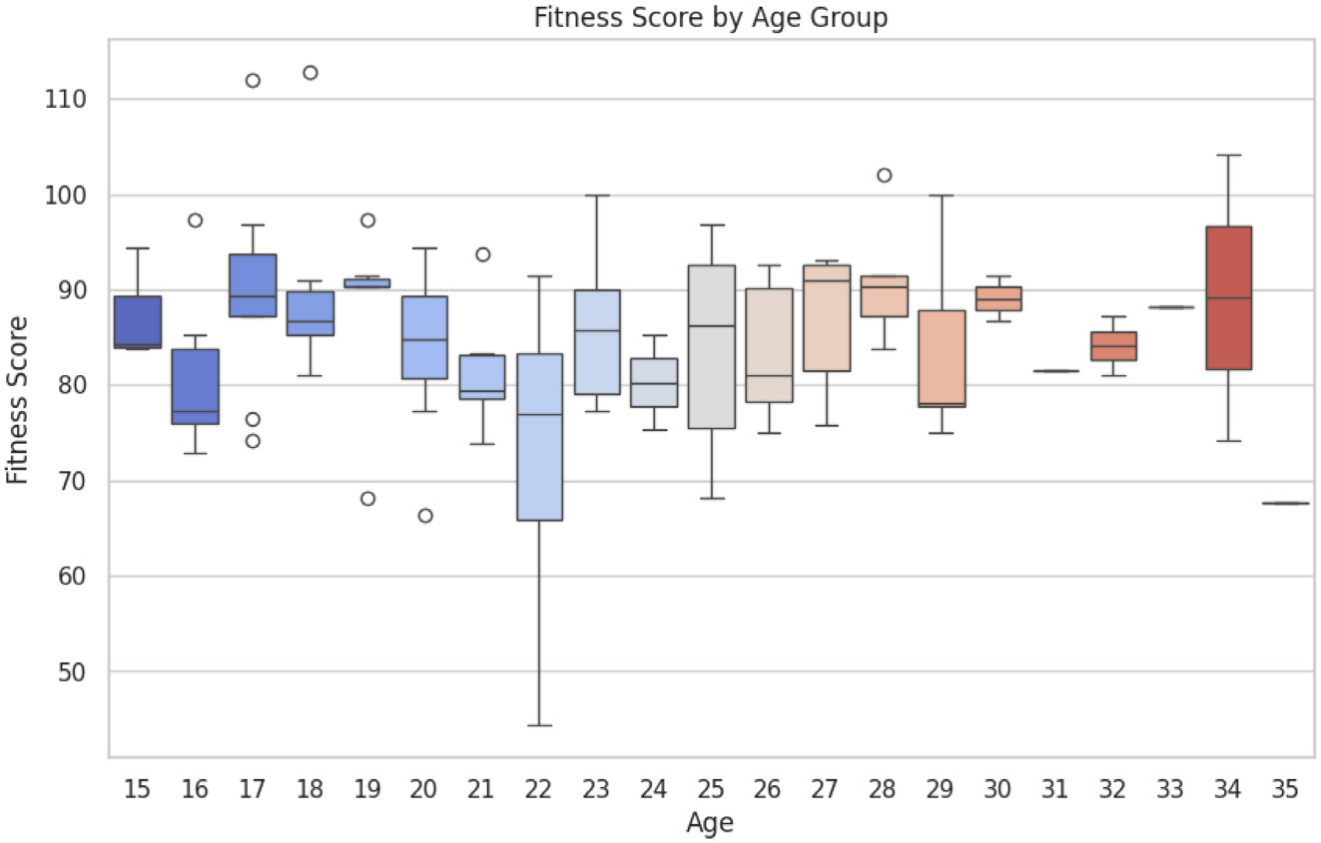
Box Plot of Fitness Score by Age.
• This heatmap Fig. 4 visualizes the correlations between numeric variables in the dataset, such as BMI, resting heart rate, fitness score, and pulse recovery rates. It helps identify strong relationships between variables, providing a clear view of how they interact with each other.

**Fig. 4:**
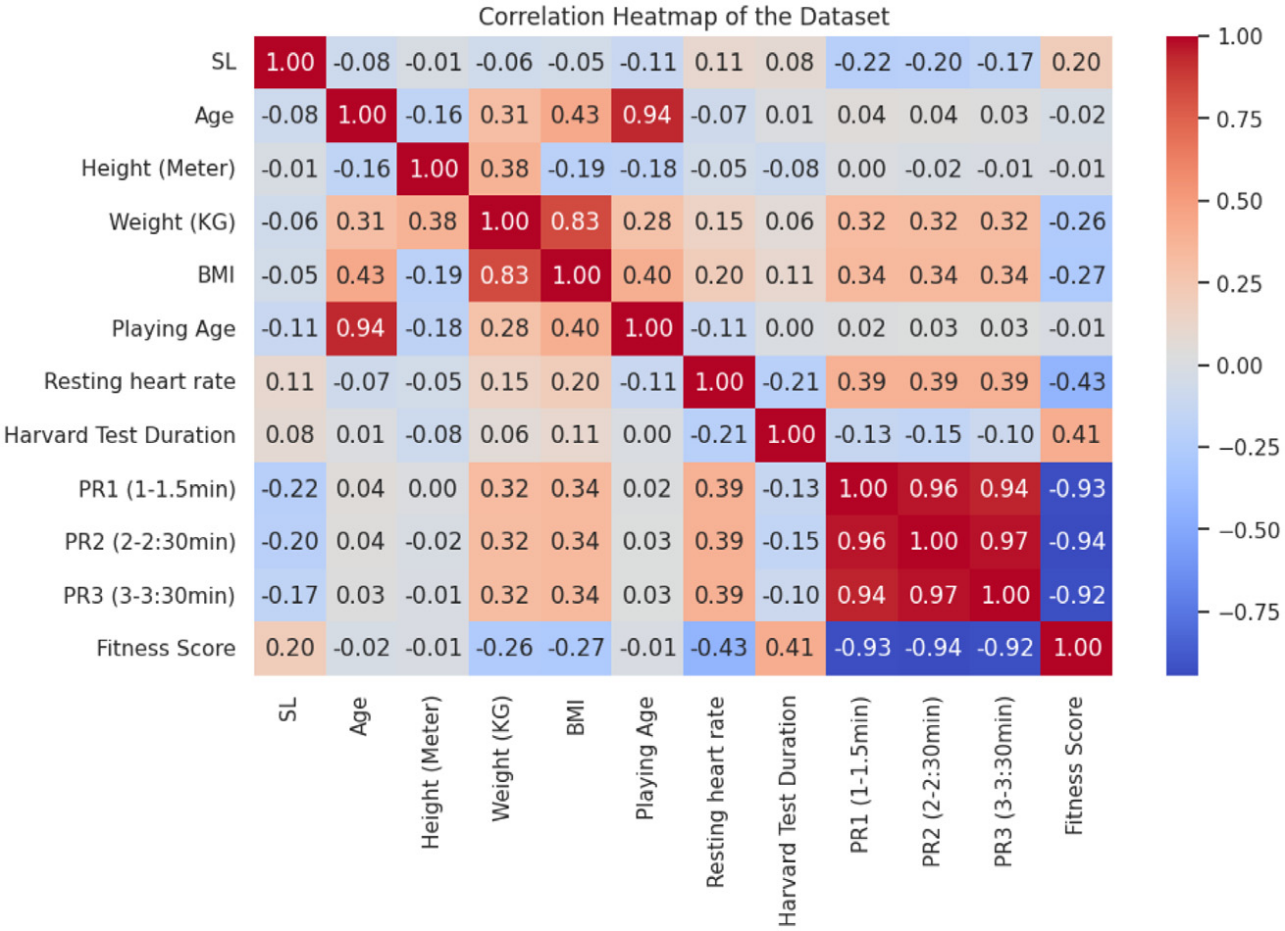
Correlation Heatmap of Variables.
• A **pair plot** Fig. 5 visualizing the pairwise relationships between selected variables such as age, BMI, resting heart rate, fitness score, and pulse recovery rates. This plot helps to examine how these variables interact and potentially identify multivariate patterns or trends.

**Fig. 5:**
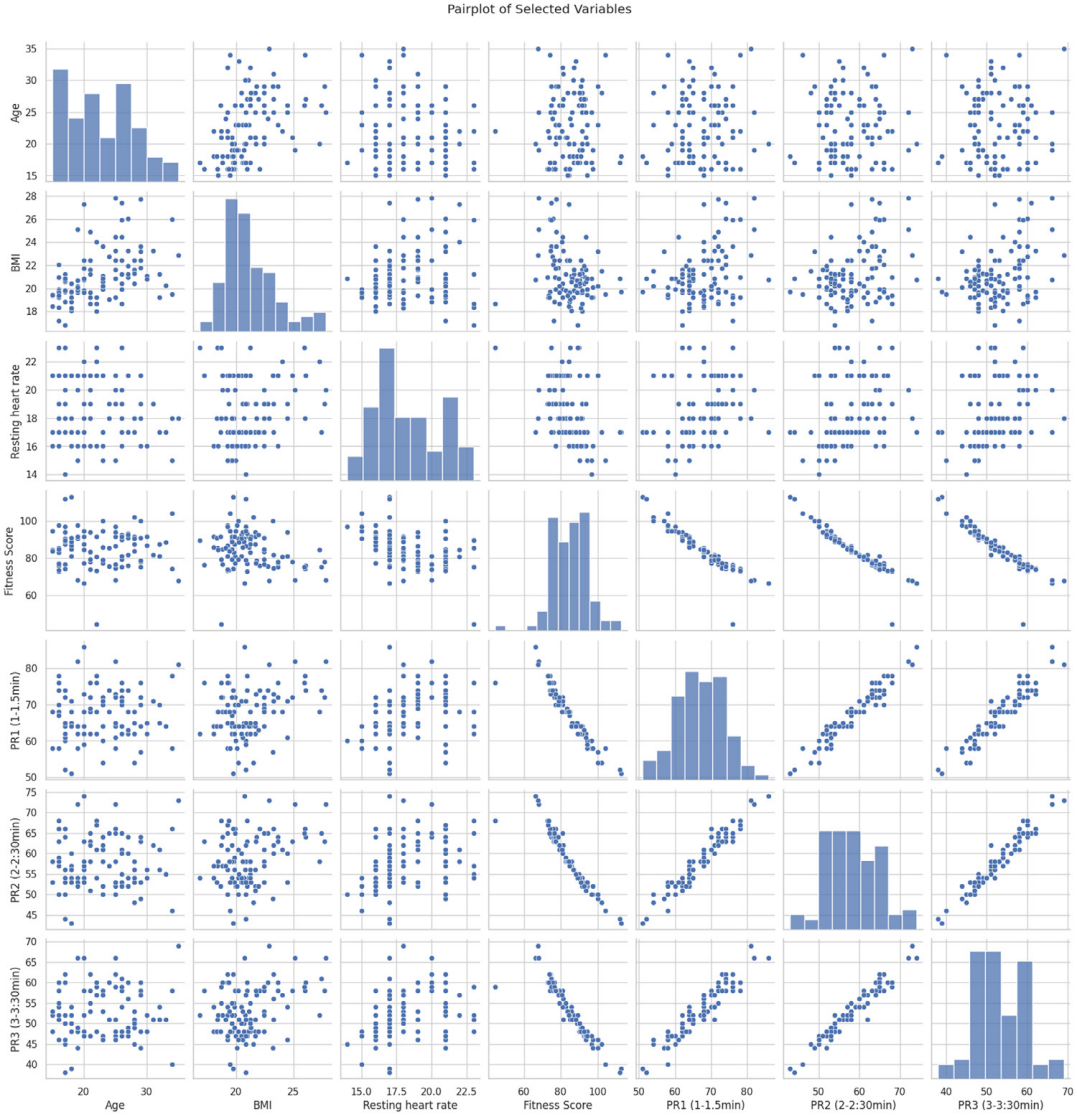
Pairplot of Selected Variables.
• This **box plot** Fig. 6 detects outliers in the resting heart rate of the participants. It helps visualize the spread of resting heart rates and highlights extreme values, which could be indicative of potential anomalies or special cases in the data.

**Fig. 6:**
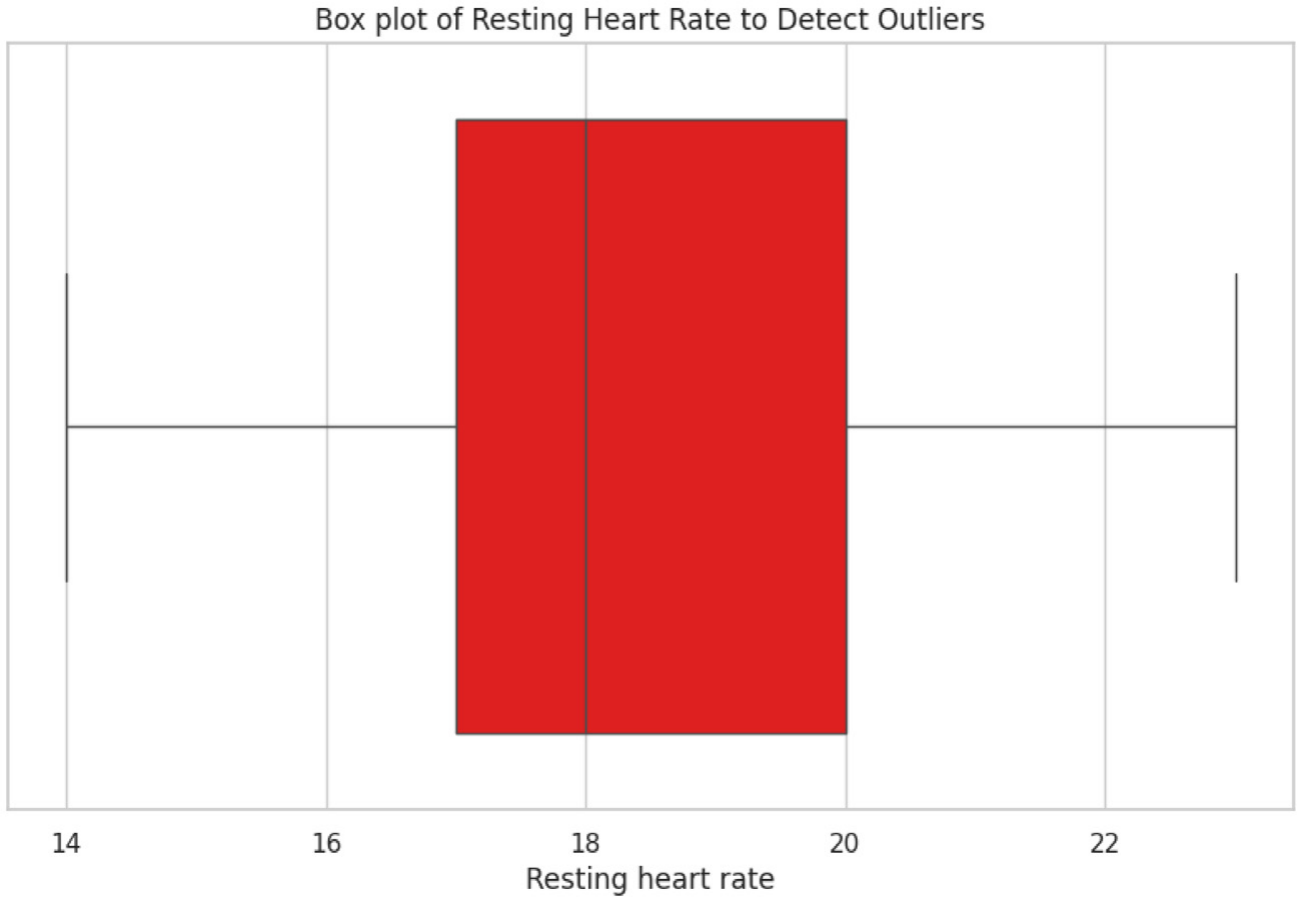
Box Plot of Resting Heart Rate.

## 4 Experimental Design, Materials and Methods

### 4.1 Participant Preparation

Participants were selected from female cricketers competing in the Bangladesh National Women's Cricket League 2021–22, the highest-level competition for women cricketers in Bangladesh. Before the test, participants' age, height, weight, and resting heart rate were recorded.

**Warm-Up**: Each participant performed a short, individual warm-up before the test following the measurement of their resting pulse rate.

**Ethical Compliance**: Ethical approval was obtained from the relevant authorities, and written consent was acquired from all participants.

### 4.2 Experimental Setup

The Harvard Step Test (HST) was conducted in a controlled environment to ensure accuracy and consistency.

**Step Platform**: A wooden bench measuring 16 inches in height, 24 inches in width, and 30 inches in length was used.

**Step Rate**: A stepping rhythm of 30 steps per minute (one step every two seconds) was maintained using a metronome app (set at 120 beats per minute).

**Duration**: Participants performed the stepping exercise for a maximum of 5 minutes or until they could no longer maintain the set pace.

### 4.3 Pulse Rate Measurement

Pulse rates were recorded at specific intervals to measure recovery.

Resting Heart Rate: Measured for one minute before the test.

### 4.4 Post-Test Recovery Pulse

PR1: Recorded between 1:00 and 1:30 minutes after the test.
PR2: Recorded between 2:00 and 2:30 minutes after the test.
PR3: Recorded between 3:00 and 3:30 minutes after the test.

### 4.5 Fitness Score Calculation

The Harvard Step Test Recovery Index (fitness score) was calculated using the formula:

$$\text{Fitness}\;\text{Score}=\;\left(\begin{array}{c} \begin{array}{c} \text{Duration}\;\text{of}\;\text{exercises}\;\left(\text{sec}\right)\;\times \;100\;\\ ------------ \end{array}\\ \text{Sum}\;\text{of}\;\text{PR}1,\;\text{PR}2,\;\text{PR}3 \end{array}\right)$$

Where:

Duration of exercise: Time (in seconds) that the participant was able to maintain the set stepping pace.

PR1, PR2, PR3: Recovery pulse rates recorded at the specified intervals.

### 4.6 Procedure

Briefing and Demonstration: Participants were briefed about the HST procedure and given a clear demonstration. Any questions or doubts were clarified before the test.

Resting Pulse Measurement: Participants were seated calmly for 5 minutes before their resting heart rate was measured.

Conducting the HST: Participants performed stepping motions on the platform, guided by the metronome, for up to 5 minutes. Volunteers provided minute-wise updates during the test.

Post-Test Recovery Pulse Measurement: Immediately after the test, participants were seated comfortably, and pulse rates were measured at the specified intervals.

Completion Criteria: Participants who maintained the pace for the full duration were recorded as completing 5 minutes. For others, the time elapsed until the test was stopped (by the participant or the investigator) was recorded.

### 4.7 Data Collection

Data was collected over three months (January–March 2022) by eight trained coaches, each representing a division in Bangladesh. Coaches were responsible for ensuring consistent administration of the test and accurate data recording.

**Tools and Materials:**

**Table:** 

Tool/Material	Description
Step Platform	Wooden bench measuring 16 inches (height), 24 inches (width), 30 inches (length).
Stopwatch	Casio 100 memory digital stopwatch for monitoring duration and stepping pace.
Metronome App	Android-based app set at 120 beats per minute to guide stepping rhythm.
Pulse Measurement	Manual pulse measurement taken from the carotid artery or using pulse oximeters.
Height & Weight Tools	Stadiometer and digital weighing scale for measuring height and weight.
Software	Microsoft Excel for data entry; Python (Pandas, Matplotlib, Seaborn) for analysis and visualization.

### 4.8 Data Analysis Methodology

The data was collected by the coaches, and all records were digitized into a standard **Excel spreadsheet**. After data collection, the following analyses were performed:

1. **Data Cleaning**: Any erroneous or missing data were handled using standard data cleaning techniques, including imputation for missing values where appropriate.
2. **Correlation Analysis**: To identify the relationships between variables (e.g., BMI, resting heart rate, and fitness score), **Pearson correlation coefficients** were calculated.
3. **Outlier Detection**: **Boxplots** and **z-scores** were used to identify potential outliers in the dataset, particularly in variables like BMI, pulse recovery rates, and fitness scores.
4. **Statistical Analysis**: Statistical tests (e.g., ***t*-tests** or **ANOVA**) were applied to compare fitness scores across age groups and to assess the impact of variables like **playing age** on fitness.
5. **Visualization**: Graphical representations, such as **scatter plots** and **histograms**, were used to visualize the distribution of variables (e.g., BMI distribution, pulse recovery trends, and fitness scores).

### 4.9 Data Quality Control

• **Consistency Check**: The dataset underwent regular consistency checks to ensure that all pulse rates were measured within the expected time frames and that the fitness scores were calculated accurately.
• **Reproducibility**: All procedures were standardized, and coaches followed strict protocols to ensure reproducibility and uniformity across all testing sites.

### 4.10 Data Storage and Sharing

The dataset is stored in a public repository (e.g., **Mendeley Data**), where it can be accessed for further analysis or research. A **README file** is included with the dataset, explaining the structure of the data, the variables, and the steps taken to ensure data integrity.

## Limitations

The dataset may have some limitations that could affect its generalizability. First, the data collection was restricted to female cricketers participating in the Bangladesh National Women's Cricket League 2021–22, and thus, the sample may not be fully representative of all female athletes or cricketers in Bangladesh. Secondly, the dataset is limited by the nature of the Harvard Step Test, which focuses primarily on cardiovascular endurance and may not capture other aspects of athletic performance, such as strength or agility. Additionally, some variables such as playing age and BMI could have measurement biases due to the self-reporting nature of certain attributes, and the data collected represents a snapshot of fitness at a single point in time. The sample size may also be considered relatively small for more extensive analysis across diverse groups. These limitations should be taken into account when interpreting the findings and drawing broader conclusions.

In our future research, we will integrate an Optimized Artificial Neural Network (ANN) [8] [[10], [11], [12]] and a Fuzzy Set approach [7,9] to systematically rank the factors within this dataset for advanced analysis. This methodological framework builds upon the seminal works of, ensuring a rigorous and evidence-based approach to factor evaluation.

## Ethics Statement

This study involved human subjects, and all procedures were carried out in accordance with the Declaration of Helsinki. Informed consent was obtained from all participants prior to data collection.

## Credit Author Statement

**Farjana Akter Boby**: Conceptualization, Data Collection, **Marzia Ahmed**: Methodology, Writing- Original draft preparation, Visualization, Writing- Reviewing and Editing. **Imran Mahmud** Investigation, Supervision, Writing- Reviewing and Editing.

## Data Availability

Mendeley DataHarvard Step Test Dataset for Female Cricketers in the Bangladesh National Women's Cricket League 2021-22 (Original data).
